# Hearing Aid Use Time Is Causally Influenced by Psychological Parameters in Mildly Distressed Patients with Chronic Tinnitus and Mild-to-Moderate Hearing Loss

**DOI:** 10.3390/jcm11195869

**Published:** 2022-10-04

**Authors:** Benjamin Boecking, Stamatina Psatha, Amarjargal Nyamaa, Juliane Dettling-Papargyris, Christine Funk, Kevin Oppel, Petra Brueggemann, Matthias Rose, Birgit Mazurek

**Affiliations:** 1 Tinnitus Centre, Charité—Universitätsmedizin Berlin, 10117 Berlin, Germany; 2 Terzo Institute, ISMA AG, 96515 Sonneberg, Germany; 3 Division of Psychosomatic Medicine, Charité—Universitätsmedizin Berlin, 10117 Berlin, Germany

**Keywords:** hearing aids, usage time, use time, mild-to-moderate hearing loss, tinnitus-related distress, psychological epiphenomena

## Abstract

Background: Hearing aids (HAs) can improve tinnitus-related distress (TRD) and speech-comprehension (SC) in silence or at 55 dB noise-interference (SC_55 dB) in patients with chronic tinnitus and mild-to-moderate hearing loss. However, the role of HA use time in relation to psychological, audiological, or self-reported tinnitus characteristics is under-investigated. Methods: We examine 177 gender-stratified patients before (t_1_) and after an intervention comprising binaural DSL_child_ algorithm-based HA fitting and auditory training (t_2_) and at a 70-day follow up [t_3_]. HA use time was retrospectively retrieved (at t_2_) for the pre-post- and (at t_3_) post-follow up periods. General linear models investigated HA use time in relation to (1) general audiological, (2) tinnitus-related audiological, (3) tinnitus-related self-report, and (4) distress-related self-report indices before and after treatment, where applicable. Receiver operator characteristic analyses identified optimal HA use time for hereby-mediated treatment changes. Results: At t_1_ and t_2_, psychological, but not audiological indices causally influenced prospective HA use time—except for SC_55 dB at t_1_, which, however, correlated with patients’ anxiety, depressivity, and psychological distress levels. Correlations did not differ between patient subgroups defined by categorical tinnitus-related audiological or self-report indices. HA use time partly mediated treatment-related improvement in TRD, but not SC. Optimal use amounted to 9.5–10.5 h/day. Conclusions: An awareness of psychological influences may help clinicians facilitate HA use and, thereby, TRD improvement with hearing amplification.

## 1. Introduction

Tinnitus denotes “the conscious awareness of a tonal or composite noise for which there is no identifiable corresponding external acoustic source” [1]. While psychological, audiological, or medical factors can facilitate tinnitus onset or maintenance, hearing loss (HL) is an important risk factor for many—though not all—tinnitus presentations [2,3,4]. Accordingly, current guidelines suggest the provision of hearing aids (HAs) as first-line intervention for individuals with HL and chronic tinnitus, alongside psychological interventions for those who experience high levels of psychological distress preceding or following symptom onset [5]. 

Both HL [6] and chronic tinnitus can contribute to difficulties with speech comprehension (SC), especially in contexts involving noise distractors [7]. Initial evidence suggests that HA use may benefit SC over time [8,9], potentially through individual levels of hearing loss linearly influencing HA use as a mediator of benefit [10]. However, neuropsychological mechanisms underlying these effects are likely complex [7,11,12,13,14,15,16,17], and research findings in this regard are limited to date. 

Despite its putative importance and comparatively easy influenceability, research at the junction of HA use time and associated psychological influences in adults is sparse [18]. The majority of studies focuses on audiological predictors of HA use [19] oand psychological influences on HL that are either unsusceptible to HA use [20] or improve following hearing amplification [21]. “Previously identified psychological predictors of HA nonuse include ‘perceived stigma’, ‘cosmetic concerns’, ‘disappointment with HA’, ‘oversold expectations’, or ‘family pressure to get HAs’ [22]. By contrast, HA use is influenced by ‘[positive] attitudes towards HAs’, ‘[realistic] expectations of benefit’, and individuals’ ‘perception- and acceptance of their hearing difficulties’ [23].” Only one study specifically examines the impact of psychological factors on HA use time - and reported a negative association between depressivity and HA use time [24]. Dawes et al. [25], however, failed to find such an association in a large cross-sectional sample.

Against the background of interacting influences of HL, chronic tinnitus symptomatology, psychological distress, and SC difficulties, few studies have investigated the effectiveness of HAs on tinnitus-related distress (TRD) or SC in silence or noise in patients with chronic tinnitus and mild-to-moderate HL. Two recent studies from our group aimed to fill this gap and reported beneficial effects of a 21-day hearing therapy on TRD [26] and SC in silence for patients with mild or moderate, and 55 dB noise-interference for patients with mild HL only [27]. Treatment involved binaural Desired Sensation Level (DSL)_child_ algorithm-based HA fittings and auditory self-study training. At 65 dB noise-interference, SC did not improve with treatment in either patient group. 

Expanding these investigations, the present study has two aims: First, to examine psychological distress levels across general audiological (hearing ability, speech comprehension in silence and at 55 dB or 65 dB noise-interference), tinnitus-related audiological (tinnitus type, location, pitch), and tinnitus-related self-report data (perceived pitch, onset, duration, as well as perceived fluctuations of sound and loudness). Second, to examine HA use time in relation to these four variable groups and herewith-associated treatment benefits on TRD or SC, respectively. We hypothesized that both audiological and psychological variables would influence HA use time and, thereby, the intervention’s benefit. 

## 2. Materials and Methods

### 2.1. Participants 

Expanding on the above-reported results [26,27,28], we use data from the original randomized controlled crossover study that investigated the effects of a hearing therapy protocol on TRD and SC. The present study examines pooled data from the crossover study’s two intervention arms and includes *N* = 177 patients with chronic tinnitus and mild-to-moderate HL (age_mean_ = 59.61 years; *SD* = 7.46) who were examined at screening (t_0_), pre- and post-treatment (t_1_ − t_2_), and at a 70-day follow up timepoint (t_3_) (see also [27]). The study was conducted according to the principles of the Declaration of Helsinki and approved by the Charité’s Ethics Committee (EA1/114/17). 

### 2.2. Data and Measures

Briefly, obtained data comprised four groups of variables: (1) general audiological data (hearing ability [Pure-Tone-Audiometry, PTA, t_0_]; SC in silence and at 55 and 65 dB noise-interference, t_1_, t_2_, t_3_); (2) tinnitus-related audiological data (tinnitus type, location, pitch, t_0_); (3) tinnitus-related self-report indices (perceived pitch, onset, duration, as well as perceived sound-and loudness fluctuations, t_0_); and (4) distress-related self-report data (Tinnitus Questionnaire, TQ, [29]; Tinnitus Handicap Inventory, THI [30]; Tinnitus Functional Index, TFI [31], Perceived Stress Questionnaire, PSQ [32]; Hospital Anxiety and Depression Scale, HADS [33]; and ICD-10 Symptom Rating, ISR [34,35], t_1_, t_2_, t_3_). 

Overall, the sample was characterized by low-to-mild (TFI) or mild-to-moderate levels of TRD (TQ, THI), respectively; normal levels of perceived stress (PSQ), anxiety, and depression (HADS), and mildly elevated general psychological distress (ISR). 

#### 2.2.1. Hearing Therapy 

The hearing therapy combined binaural DSL_child_ algorithm-based HA fittings and a 14-day auditory self-study program (terzo^©^ Hearing Therapy). For detailed information on sample characteristics at screening [28] as well as study design, sample characteristics at baseline, the examined hearing therapy, and the obtained self-report measures, readers are referred to the current study’s predecessor papers [26,27].

#### 2.2.2. Hearing Aid Use Time 

The present study used Mood 16 G4 HAs. HA use time (h/day) was retrospectively retrieved (at t_2_) for the pre-post- and (at t_3_) for the post-follow up periods, thus allowing for a causal interpretation of correlation coefficients at pre- or post-treatment respectively.

### 2.3. Statistical Analyses

First, descriptive analyses and univariate comparisons (independent-samples *t* tests and analyses of variance, ANOVAs) examined tinnitus-related audiological and tinnitus-related self-report indices relative to general audiological- and distress-related self-report variables. 

Second, Pearson correlation coefficients *r* investigated (1) associations between general audiological as well as distress-related self-report data at pre- and post-treatment and HA use time, as well as (2) possible differences in any such associations for patient subgroups who differed on factors identified in Step 1. Here, similar to our approach in [27], coefficients were compared using MedCalc (https://www.medcalc.org/calc/comparison_of_correlations.php; accessed on 19 August 2022), where applicable. Correlational effects were interpreted according to Cohen [36] (*r* ≥ 0.10 = small effect, *r* ≥ 0.30 = moderate effect, *r* ≥ 0.50 = strong effect). 

Third, Hayes’ PROCESS macro [37] calculated simple mediation models that examined ‘true’ mediation [38] of pre (x)-to-post (y)-treatment changes in SC or distress-related variables via (retrospectively quantified) HA use time (m). For significant indirect effects, Receiver operator characteristic (*ROC*) analyses further aimed to quantify the optimal HA use time associated with treatment-related ‘improvement’ (vs. ‘no improvement’), pragmatically defined as any pre-to-post-treatment change to the positive (SC) or negative (TQ, THI, TFI, PSQ, HADS_a, HADS_d, ISR), respectively. Here, the ‘area under the curve’ statistic (*AUC*) reflects HA use time’s poor (0.50 < *AUC* < 0.70), acceptable (0.71 < *AUC* < 0.90), or outstanding ability (*AUC* > 0.91) to perform this distinction [39,40]. 

All analyses were computed using SPSS statistical software version 27 (SPSS Inc., Chicago, IL, USA). Of note, analyses revealed no significant effects for the post- to follow up period—likely owing to the relative stability of all treatment-related effects (cf. [26,27]). The present paper thus limits itself to reporting findings for the t_1_-t_2_ intervention period. 

## 3. Results

### 3.1. Tinnitus-Related Audiological and Tinnitus-Related Self-Report Indices in Relation to General Audiological and Distress-Related Self-Report Data 

Table 1 reports between-group differences in general audiological- (Panel a) and distress-related (Panels b and c) variables across categorical tinnitus-related audiological and tinnitus-related self-report indices, where applicable. 

Results revealed that patients’ PTA-measured hearing ability was lower for patients reporting previous hearing aid use and gradual tinnitus onset. 

SC in silence was aggravated for patients reporting previous hearing aid use and narrow-band tinnitus perception. At medium noise-interference (SC_55 dB), patients with a history of psychotherapeutic support reported higher SC difficulties. At 55 and 65 dB noise-interference, higher SC difficulties were further accompanied by a ‘very high’ (vs. high) self-reported tinnitus pitch. 

Significantly higher levels of psychological distress were reported by patients who were female (TFI, PSQ, HADS_a, ISR), had a history of psychotherapeutic support (TQ, THI, TFI, PSQ, HADS_a, HADS_d, ISR), described a ‘very high’ (vs. middle: TQ, HADS_a; or vs. high vs. middle: THI, TFI, HADS_d, ISR) self-reported tinnitus pitch, reported sudden tinnitus onset (THI, PSQ), experienced no intermittence (TFI), and reported fluctuations in perceived loudness (PSQ, HADS_a). 

The majority of patients reported a ‘high’ tinnitus pitch. Yet, despite comparable proportions of patients in PTA-measured vs. self-reported tinnitus frequency ranges, statistical agreement between the two variables was only “slight” (Cohen’s κ = 0.12; *p* < 0.05, [41]), indicating an importance of independent measurement and conceptualization. 

### 3.2. Hearing Aid Use Time and General Audiological, Tinnitus-Related Audiological, Tinnitus-Related Self-Report-, and Distress-Related Self-Report Data 

Participants’ average daily HA use time amounted to 9.26 (*SD* = 4.14) for the t_1_−t_2_ period and 9.49 (*SD* = 4.25) h for the t_2_−t_3_ period, respectively. It did not differ between any patient subgroups who were characterized by differences in categorical tinnitus-related audiological or tinnitus-related self-report indices.

Table 2 reports Pearson’s *r* correlations between general audiological as well as distress-related self-report indices and subsequent HA use time. At pre-treatment, small-to-moderate causal effects emerged for psychological, but not audiological variables. An exception was found for SC_55 dB, which was further associated with both patients’ hearing ability, *r* = −0.40, *p* < 0.001 (‘moderate’), and indices of anxiety, *r* = −0.18, *p* < 0.05; depression, *r* = −0.20, *p* < 0.01; and general psychological-, but not tinnitus-related distress, *r* = −0.26, *p* < 0.01 (‘small’). At post-treatment, psychological variables continued to causally influence prospective HA use time during the follow up period in the small-to-moderate range.

SC in silence and at 65 dB noise-interference did not influence HA use time. SC_0 dB was associated with patients’ hearing ability, *r* = −0.19, *p* < 0.05, TRD (THI: *r* = −0.16, *p* < 0.05; TFI: *r* = −0.19, *p* < 0.05) and perceived stress, *r* = −0.17, *p* < 0.05 (‘small’). SC_65 dB was associated with patients’ hearing ability, *r* = −0.28, *p* < 0.001, depression, *r* = −0.17, *p* < 0.05, and general psychological distress, *r* = −0.17, *p* < 0.05 (‘small’). 

Linking findings from Section 3.1 and Section 3.2, additional analyses investigated, whether correlation coefficients between HA use time and influencing parameters (cf. Table 2) differed between patient subgroups who were characterized by differences in categorical tinnitus-related audiological or tinnitus-related self-report indices (cf. Table 1). For example, because (1) TQ-measured TRD causally influenced subsequent HA use time (cf. Table 2), and (2) TQ scores significantly differed for participants with vs. without previous psychotherapy (cf. Table 1), correlation coefficients *r*_TQ HA use time_ were compared between these patient subgroups. 

Overall, results revealed no between-subgroup differences in correlational strengths. An exception was found for *r*_SC_55 dB_HA use time_, which only emerged in patients with a ‘high’, *r* = −0.31, *p* < 0.01 (but not ‘very high’, *r* = 0.10, *n.s.*) tinnitus pitch (*z* = 2.07, *p* < 0.05). 

### 3.3. Mediation Analyses 

Simple mediation analyses examined effects of HA use time (m) on treatment-related changes in SC and distress-related variables between t_1_ (x) and t_2_ (y). Results indicated that HA use time partly mediated pre- to post-treatment change in TRD as measured by the TQ (path a: −0.07, SE = 0.02, *p* < 0.001; path b: −0.36, SE = 0.16, *p* < 0.05; ab = 0.03, SE = 0.02) and TFI (path a: −0.05, SE = 0.015, *p* < 0.001; path b: −0.85, SE = 0.28, *p* < 0.01; ab = 0.05, SE = 0.02). Here, higher TRD levels at baseline negatively affected subsequent HA use time and, thereby, TRD-related improvement with treatment. By contrast, HA use time did not mediate changes in THI scores, SC indices, or other distress-related variables. 

#### Receiver Operator Characteristics Analyses

Following up on the identified indirect effects, ROC analyses aimed to identify the optimal HA use time that distinguished pre- to post-treatment ‘improvement’ (from ‘no improvement’) on the TQ or TFI. While point estimates were not significant, trend significant AUC statistics within poor-to-acceptable confidence intervals suggested minima of 9.5 (TQ; 0.47–0.75, *p* < 0.10) and 10.5 h/day respectively (TFI; 0.48–0.77, *p* < 0.10).

## 4. Discussion

The present study demonstrated that HA use time (1) is causally influenced by psychological parameters and (2) partly mediates tinnitus distress-related, but not speech comprehension improvements in mildly distressed patients with chronic tinnitus and mild-to-moderate hearing loss.

One-hundred seventy-seven gender-stratified patients with chronic tinnitus and mild-to-moderate HL were binaurally fitted with DSL_child_ algorithm-based HAs and completed auditory training exercises over a 21-day period. Measurements in TRD, anxiety, depressivity, general psychological distress, and SC in silence as well as at 55 or 65 dB noise-interference were obtained at screening (t_0_), before (t_1_) and after the intervention (t_2_), and at a 70-day follow up (t_3_). Previously published studies that examined this dataset reported controlled improvements in TRD (TQ, THI, TFI) alongside uncontrolled small improvements in anxiety and psychological distress levels (HADS_a, ISR) [26], as well as HA-related improvements in SC in silence (for patients with mild or moderate HL) and at 55 dB noise-interference (for patients with mild HL only) [27]. 

### 4.1. Patients’ Self-Report and Audiological Data 

First, the present study examined differences in general audiological ([PTA-measured] hearing ability, SC) or psychological distress indices (TQ, THI, TFI, PSQ, HADS, ISR) across patient subgroups characterized by tinnitus-related audiological (tinnitus type, location, pitch) or tinnitus-related self-report indices (perceived tinnitus pitch, onset, duration, as well as perceived sound- and loudness fluctuations). 

Here, self-reported ‘sudden’ tinnitus onset was associated with proportionately higher levels of perceived stress and THI-measured TRD. Previous research has highlighted links between sudden tinnitus and ‘stress’ or, relatedly [42,43], sudden hearing loss in patients’ own tinnitus narratives [44] as well as emotional difficulties in patients with experiences of traumatization [45]. By contrast, a reported history of ‘gradual’ onset was associated with lower PTA-measured hearing ability. For some patients, gradually developing hearing loss might parallel the perception of tinnitus [46], emphasizing a need for preventative or early-onset hearing protection measures that might delay both clusters of difficulty [47,48,49] alongside associated broader emotional difficulties [50,51]. 

The dissociation between self-reported sudden vs. gradual tinnitus onset and observed psychological vs. hearing ability-related influences may reflect a particular importance of stress-related factors for the former type of onset [52,53], particularly within a broader psychological context of pre-existing vulnerability [54,55]. For the chronification or maintenance of TRD, however, psychological factors may contribute to the appraisal of the tinnitus sound regardless of onset trajectory, potentially explaining varying TRD levels across both psychologically or audiologically mediated onset patterns [56]. 

Moreover, patients with higher levels of perceived stress and anxiety reported fluctuations in perceived tinnitus loudness, and patients with higher psychological distress levels or SC-in-noise difficulties reported a ‘very high’ tinnitus pitch. In keeping with some previous findings, audiometric frequency matching did not mirror this association [57,58]. Thus, rather than high-pitched noise being perceived as aversive, psychological distress likely shapes the appraisal and experience of the tinnitus sound [59]. Previous research has suggested ‘emotional tension’ or ‘worry’ as transdiagnostic factors that potentially underlie TRD [60]. Because patients’ emotional states likely mediate the appraisal and experience of the tinnitus sound [61,62], it is crucially important to understand and conceptualize patients’ distress experiences holistically, i.e., beyond the influence of the tinnitus symptom [63]. Any such accounts, however, are necessarily complex and idiosyncratic, thus necessitating person- (not symptom-) focused psychological formulations and treatment plans [64,65,66]. Clinically, patients who report sudden tinnitus onset or loudness fluctuations may particularly benefit from clinicians’ awareness and consideration of psychological influences beyond tinnitus as the presenting index symptom, as well as their own emotional reactions to respective patient presentations [67,68,69,70]. Ideographic associations between patients’ psychological distress levels and experienced characteristics of the tinnitus sound remain uninvestigated.

Patients’ PTA-measured hearing ability correlated moderately with their SC abilities. Interestingly, SC_55 dB further correlated with patients’ anxiety, depressivity, and general psychological, but not tinnitus-related distress levels. By contrast, SC_0 dB yielded a roughly inverse pattern. Moreover, SC_55 dB was lower in patients with a history of psychotherapeutic support, who further reported higher levels of distress across all psychological indices. 

Patients with chronic tinnitus commonly report difficulties with SC, which can (but does not have to) be associated with hearing difficulties, potentially reflecting a ‘functional’ component in some patients [71]. Psychologically, SC is underlain by a multitude of cognitive processes such as inhibitory control, processing speed, allocation of attentional resources, or working memory [72,73], all of which are also known to interact with affective states such as anxiety or mood [13,74,75,76,77,78,79,80,81,82]. In a recent study, Tai and Husain [83] suggested that SC in noise may be influenced by interactions of ongoing tinnitus perception, cognitive control of emotion (involving the perception of, orientation towards, appraisal of, and reaction to the tinnitus sound), and cognitive control of attention. 

Speculatively, SC might follow an inverse U-curve characterized by inversely proportional ratios of hearing- vs. emotion-related influences under circumstances of increasing noise-interference [84,85,86,87], with emotion-related influences reaching their proportionate maximum at medium noise-interference. Future studies might wish to test this possibility by measuring patients’ SC across linearly increased noise-interference levels in patients at varying levels of HL and psychological distress. 

In keeping with previous findings, female patients reported higher levels of tinnitus-related [88,89,90,91] and general psychological distress [92,93,94,95,96]. Studies aiming to explain this gender discrepancy suspect the existence of gender-specific (hormonal [97]) phenotype clusters [98] or high numbers of emotionally stressed men who do not access available support options, potentially influenced by masculine gender norms [99,100,101,102,103,104]. 

Moreover, intermittent perception of the tinnitus sound was associated with lower levels of TFI-measured TRD, supporting some [105,106], but not all previous findings [107]. Underlying factors likely include both cognitive or behavioral processes such as higher attentional control [108], or individuals’ distress-related (in)abilities to distract themselves from the tinnitus percept [56,109]. Alternatively, however, the finding may reflect an artifact owed to some of the TFI’s item phrasings (e.g., “What percentage of your time awake were you consciously aware of your tinnitus?”). 

### 4.2. Hearing Aid Use Time

Second, we examined the four obtained variable groups (general audiological, tinnitus-related audiological, tinnitus-related self-report, and distress-related self-report indices) in relation to HA use time and associated treatment benefit. Owing to the retrospective retrieval of HA use time, correlation coefficients could be interpreted causally. Results revealed small yet significant causal influences of both tinnitus-related and broader psychological distress on HA use time at both pre- and post-treatment. 

Relatedly, HA use time partly mediated treatment-related change in TRD as measured by the TQ and TFI, with higher TRD levels at baseline reducing prospective HA use time - thereby lowering treatment benefit as measured by these indices. According to Van der Wal et al. [110], the TQ captures the “psychological“, and the TFI the “body functions” and “activity and participation”-related impact of chronic tinnitus symptomatology. A similar suggestion was made by Boecking et al. [111], who discussed “psychological” vs. “audiological” characteristics of TRD as measured by the TQ or TFI, respectively. Associations between pre-existing psychological distress, HA use, HA use time, and subsequent psychological, hearing-related or participation-based benefits are, however, likely bidirectional and closely interrelated. Notwithstanding, while HA-related benefits on TRD have been previously demonstrated in patients with chronic tinnitus and HL [5,112,113,114,115], our study is the first to demonstrate a vicious cycle wherein TRD at baseline likely decreases the use of the very intervention likely to benefit it.

Supplementary analyses revealed at trend level that an average use time of 9.5-to-10.5 h/day best distinguished between patients who showed improvement (vs. no improvement) on the TQ or TFI, respectively. Although these results necessitate replication due to a lenient definition of ’improvement’ and rather broad confidence intervals around the AUC statistics, they do suggest that HA use time partly influences TRD improvement (in context of DSL_child_ algorithm-based HA fittings for patients with mild-to-moderate HL) – yet by no means exclusively so. Clinicians may wish to emphasize or review associations between baseline TRD, likely effects on HA use time, and resulting improvements for individuals with chronic tinnitus and mild-to-moderate HL.

By contrast, HA use did not mediate changes in anxiety, depressivity, or general psychological distress. Mirroring previous observations [116], this finding likely reflects the multifactorial, non-audiological origin and breadth of peoples’ emotional experiences [117] as well as the overall only mild distress levels in the present sample [26]. 

Interestingly, HA use time did not mediate changes in patients’ SC levels either: Neither patients’ PTA-measured hearing ability nor SC levels at 0 or 65 dB noise-interference causally influenced prospective HA use time. By contrast, SC_55 dB *did* do so; however, HA use time did not predict treatment-related change on this index—which was therefore influenced by other, unmeasured variables. We further observed indications of a double dissociation wherein SC_55 dB was associated with general psychological, but not tinnitus-related distress, and a roughly inverse pattern emerged for SC_0 dB. Future studies might wish to experimentally study the effects of people’s affective states on SC at varying levels of HL, noise-interference, or amplification.

Overall, the observed mediation pattern appears to reflect both the psycho-audiological nature of TRD in patients with chronic tinnitus and HL [5] and the clinical need to conceptualize and address psychological influences on hearing- as well as SC difficulties beyond amplification alone [118]. 

### 4.3. Limitations 

The present study has important limitations. Most notably, the interpretability and generalizability of results is inconclusive, owing to overall ‘mild’ psychological distress levels, a primarily amplification-based treatment protocol, and dual ‘index symptoms’ (chronic tinnitus symptomatology and mild-to-moderate HL) that may independently or interactionally affect both SC and psychological distress as outcomes of interest. Future studies might wish to examine chronic tinnitus patient samples with dimensionally distributed rates of hearing loss, speech comprehension difficulties, noise-interference levels, and psychological distress levels. 

### 4.4. Conclusions

In summary, the present study highlights the importance of psychological factors in motivating HA use time for patients with chronic tinnitus and mild-to-moderate HL, with direct effects on TRD-improvements following amplification-based hearing therapy. To this end, certain self-reported tinnitus characteristics may serve as tentative markers of psychological distress that ought to be conceptualized holistically within patients’ broader life contexts [54,64,119,120,121]. Clinicians might wish to counsel individuals sensitively about links between baseline TRD, HA use time, and realistically expectable amplification benefits. The influence of psychological factors on SC difficulties is currently unclear and warrants further examination, particularly in circumstances of medium noise-interference. 

## Figures and Tables

**Table 1:** Sample descriptors and univariate comparisons for general audiological (a), tinnitus-related (b), and other distress-related indices (c). PTA = pure tone audiometry; SC = speech comprehension; TRD = tinnitus-related distress; TQ = Tinnitus Questionnaire; THI = Tinnitus Handicap Inventory; TFI = Tinnitus Functional Index; PSQ = Perceived Stress Questionnaire; HADS_a = Hospital Anxiety and Depression Scale_anxiety subscale; HADS_d = depression subscale; ISR = ICD-10 symptom rating. *Italicised numbers* denote significantly differing contrasts. * *p* < 0.05; ** *p* < 0.01; *** *p* < 0.001.

General Audiological Indices				Hearing Ability [PTA]			SC_0 dB			SC_55 dB			SC_65 dB		
Descriptors		*n*	%	*M*	*SD*	*F*	*M*	*SD*	*F*	*M*	*SD*	*F*	*M*	*SD*	*F*
Gender															
	male	81	45.8												
	female	96	54.2												
Previous psychotherapy															
	no	124	70.1							73.75	13.16	(1171) = 3.96 *			
	yes	53	29.9							69.34	14.08				
Previous hearing aid use															
	no	123	69.5	35.82	7.36	(1170) = 4.89 *	98.55	2.62	(1174) = 13.57 ***						
	yes	53	29.9	40.11	6.41		95.88	12.72							
Tinnitus type															
	pure-tone	121	68.4				98.63	2.59	(1167) = 5.97 *						
	narrow-band	52	29.4				95.61	12.94							
Tinnitus location															
	right	15	8.5												
	left	31	17.5												
	both	131	74.0												
Tinnitus pitch															
	very high	-	-												
	high	104	58.8												
	middle	37	20.9												
	low	7	4.0												
Perceived tinnitus pitch															
	very high	37	20.9							*66.71*	*11.17*	(3169) = 3.11 *	*18.16*	*13.58*	(3169) = 2.72 *
	high	104	58.8							*74.28*	*14.35*		*27.55*	*20.02*	
	middle	32	18.1							72.76	12.14		21.72	17.33	
	low	3	1.7							77.50	3.54		22.50	3.54	
Perceived tinnitus onset															
	gradual	92	52.0	37.92	6.79	(1163) = 4.78 *									
	sudden	73	41.2	35.48	7.51										
Perceived tinnitus duration															
	<1/2 year	5	2.8												
	1/2–1 year	9	5.1												
	1–2 years	23	13.0												
	2–5 years	24	13.6												
	>5 years	107	60.5												
Perceived sound intermittence															
	intermittent	22	12.4												
	permanent	155	87.6												
Perceived loudness fluctuation															
	constant	71	40.1												
	variable	105	59.3												
(**a**)															
**Tinnitus-related distress indices**		**TQ**					**THI**			**TFI**					
Descriptors		*M*			*SD*	*F*	*M*	*SD*	*F*	*M*		*SD*	*F*		
Gender															
	male									36.90		19.73	(1171) = 4.03 *		
	female									43.35		22.01			
Previous psychotherapy															
	no	28.97			14.33	(1171) = 7.97 **	27.40	19.41	(1171) = 15.43 ***	38.08		19.59	(1171) = 4.96 *		
	yes	36.30			18.62		41.32	25.61		45.78		23.80			
Previous hearing aid use															
Tinnitus type															
Tinnitus location															
Tinnitus pitch															
Perceived tinnitus pitch															
	very high	*37.89*			*17.15*	(3171) = 3.76 *	*42.81*	*23.76*	(3.171) = 5.64 **	*50.85*		*24.34*	(3171) = 5.62 **		
	high	30.32			15.94		*30.25*	*21.69*		*39.23*		*20.04*			
	middle	*26.00*			*12.48*		*22.90*	*17.41*		*31.30*		*15.20*			
	low	20.00			12.73		12.00	14.14		26.86		8.57			
Perceived tinnitus onset															
	gradual						28.09	20.67	(1.160) = 5.41 *						
	sudden						36.45	24.93							
Perceived tinnitus duration															
Perceived sound intermittence															
	intermittent									30.25		18.37	(1172) = 5.67 *		
	permanent									41.85		21.23			
Perceived loudness fluctuation															
(**b**)															
**Other psychological distress-related indices**		**PSQ**					**HADS_a**			**HADS_d**			**ISR**		
Descriptors		*M*			*SD*	*F*	*M*	*SD*	*F*	*M*	*SD*	*F*	*M*	*SD*	*F*
Gender															
	male	25.33			15.37	(1171) = 6.53 *	5.50	3.81	(1171) = 4.63 *				0.52	0.45	(1171) = 4.50 *
	female	32.77			21.64		6.84	4.27					0.69	0.57	
Previous hearing aid use															
Previous psychotherapy															
	no	23.67			14.03	(1171) = 42.89 ***	5.12	3.33	(1171) = 35.09 ***	4.48	4.13	(1171) = 25.42 ***	0.48	0.38	(1171) = 29.19 ***
	yes	42.43			23.29		8.81	4.59		8.15	4.93		0.91	0.67	
Tinnitus type															
Tinnitus location															
Tinnitus pitch															
Perceived tinnitus pitch															
	very high						*7.92*	*4.83*	(3170) = 3.57 *	*7.78*	*5.52*	(3170) = 4.30 **	*0.87*	*0.61*	(3171) = 4.89 **
	high						5.99	3.92		*5.28*	*4.45*		*0.57*	*0.51*	
	middle						*4.97*	*3.20*		*3.97*	*3.63*		*0.42*	*0.35*	
	low						3.50	0.71		4.00	1.41		0.43	0.15	
Perceived tinnitus onset															
	gradual	26.64			16.56	(1160) = 4.50 *									
	sudden	33.45			22.86										
Perceived tinnitus duration															
Perceived sound intermittence															
Perceived loudness fluctuation															
	constant	25.14			16.67	(1170) = 5.52 *	5.26	3.89	(1170) = 6.58 *						
	variable	32.14			20.64		6.88	4.17							
(**c**)															

**Table 2:** Significant correlation coefficients between HA use time (t_1_ − t_2_) and general audiological as well as distress-related indices at pre- and post-treatment. Patients’ hearing ability was measured at a preceding screening timepoint. PTA = pure tone audiometry; SC = speech comprehension; TQ = Tinnitus Questionnaire; THI = Tinnitus Handicap Inventory; TFI = Tinnitus Functional Index; PSQ = Perceived Stress Questionnaire: HADS_a = Hospital Anxiety and Depression Scale, anxiety; HADS_d = depression; ISR = ICD-10 Symptom Rating; * *p* < 0.05; ** *p* < 0.01; *** *p* < 0.001.

t_1_ *n* = 155	HA use Time [t_1_ − t_2_]	t_2_ *n* = 150	HA use Time [t_2_ − t_3_]
Hearing ability [PTA]			
SC_0 dB			
**SC_55 dB**	−0.17 *		
SC_65 dB			
**TQ**	−0.30 ***		−0.32 ***
**THI**	−0.26 ***		−0.29 ***
**TFI**	−0.29 ***		−0.42 ***
**PSQ**	−0.19 *		−0.20 *
**HADS_a**	−0.17 *		−0.23 **
**HADS_d**	−0.23 **		−0.19 *
**ISR**	−0.20 *		−0.27 **

## Data Availability

As per Charité—Universitätsmedizin Berlin’s ethics committee, unfortunately, we cannot make the data public without restrictions, because we did not obtain patients’ consent to do so at the time. Nevertheless, interested researchers can contact the directorate of the Tinnitus Center at the Charité—Universitätsmedizin Berlin with data access requests (birgit.mazurek@charite.de).
